# Tetra-μ-acetato-κ^8^ *O*:*O*′-bis­[(pyridine-2-carbo­nitrile-κ*N* ^1^)copper(II)]

**DOI:** 10.1107/S1600536813034120

**Published:** 2013-12-24

**Authors:** Mei Luo, Lei Wang, Jing-Cheng Zhang

**Affiliations:** a Hefei University of Technology, Hefei, People’s Republic of China

## Abstract

The title binuclear compound, [Cu_2_(CH_3_COO)_4_(C_6_H_4_N_2_)_2_], lies about an inversion center, with the Cu^II^ cation bridged by four acetate anions and coordinated by a pyridine N atom in a distorted square-pyramidal geometry. The Cu⋯Cu distance is 2.5997 (15) Å. In the crystal, mol­ecules are linked by weak C—H⋯O and C—H⋯N hydrogen bonds into a three-dimensional supra­molecular architecture. The crystal studied was a non-merohedral twin with a minor twin component of 4.1 (1)%.

## Related literature   

For related binuclear compounds, see: Fairuz *et al.* (2010 ▶); Chang *et al.* (2011 ▶).
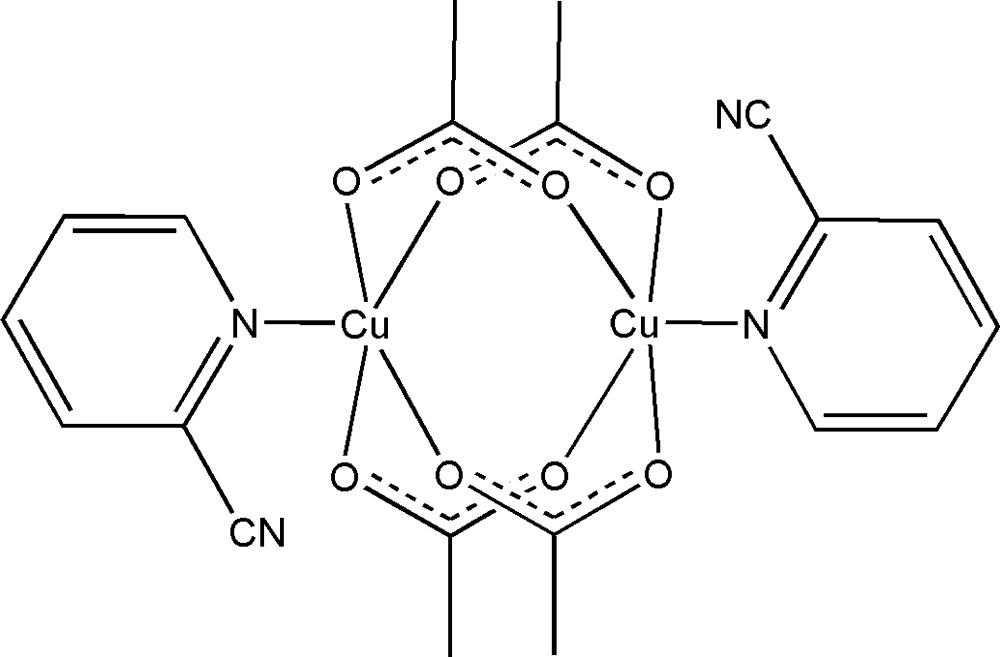


## Experimental   

### 

#### Crystal data  

[Cu_2_(C_2_H_3_O_2_)_4_(C_6_H_4_N_2_)_2_]
*M* *_r_* = 571.48
Monoclinic, 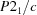

*a* = 7.929 (5) Å
*b* = 19.817 (12) Å
*c* = 8.222 (5) Å
β = 118.83 (2)°
*V* = 1131.8 (12) Å^3^
*Z* = 2
Mo *K*α radiation
μ = 1.93 mm^−1^
*T* = 140 K
0.32 × 0.12 × 0.06 mm

#### Data collection  

Bruker APEXII CCD diffractometer
Absorption correction: multi-scan (*SADABS*; Bruker, 2001 ▶) *T*_min_ = 0.58, *T*_max_ = 0.89
7361 measured reflections
2077 independent reflections
1792 reflections with *I* > 2σ(*I*)
*R*_int_ = 0.165

#### Refinement  

*R*[*F* ^2^ > 2σ(*F* ^2^)] = 0.060
*wR*(*F* ^2^) = 0.159
*S* = 1.07
2077 reflections
157 parameters
H-atom parameters constrained
Δρ_max_ = 0.89 e Å^−3^
Δρ_min_ = −1.48 e Å^−3^

### 

Data collection: *APEX2* (Bruker, 2007 ▶); cell refinement: *SAINT* (Bruker, 2007 ▶); data reduction: *SAINT*; program(s) used to solve structure: *SHELXTL* (Sheldrick, 2008 ▶); program(s) used to refine structure: *SHELXTL*; molecular graphics: *SHELXTL*; software used to prepare material for publication: *SHELXTL*.

## Supplementary Material

Crystal structure: contains datablock(s) I, global. DOI: 10.1107/S1600536813034120/xu5755sup1.cif

Structure factors: contains datablock(s) I. DOI: 10.1107/S1600536813034120/xu5755Isup2.hkl

Additional supporting information:  crystallographic information; 3D view; checkCIF report

## Figures and Tables

**Table 1:** Selected bond lengths (Å)

Cu1—O1	1.975 (4)
Cu1—O2^i^	1.980 (4)
Cu1—O3^i^	1.941 (4)
Cu1—O4	1.952 (4)
Cu1—N1	2.235 (4)

Symmetry code: (i) 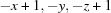.

**Table 2:** Hydrogen-bond geometry (Å, °)

*D*—H⋯*A*	*D*—H	H⋯*A*	*D*⋯*A*	*D*—H⋯*A*
C2—H2⋯N2^ii^	0.93	2.60	3.196 (9)	122
C8—H8*B*⋯N2^iii^	0.96	2.57	3.491 (8)	160
C10—H10*A*⋯O4^iv^	0.96	2.54	3.381 (7)	147

Symmetry codes: (ii) 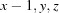; (iii) 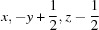; (iv) 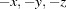.

**Fig. 1.**
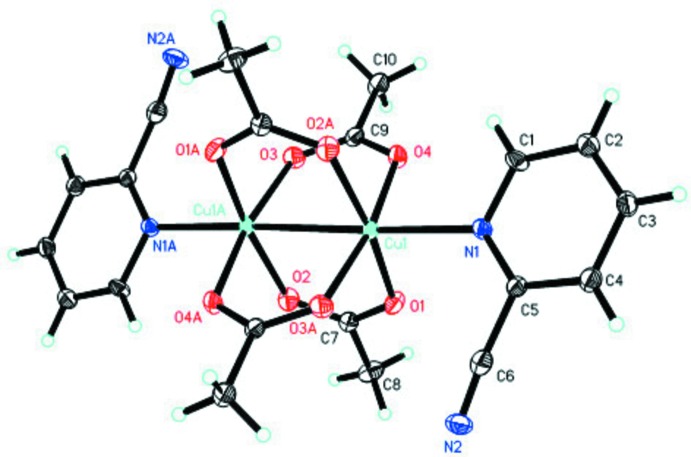
The structure of the title compound with displacement ellipsoids drawn at the 50% probability level.

## supplementary crystallographic information

### 1. Comment

The Cu–N complexes have occupied an important position in catalytic processes. In connection with on-going studies into the structural characterization of tetrakisacetatobis[(substituted 2-aminopyridyl)copper] complexes (Fairuz *et al.*, 2010; Chang *et al.* (2011), the title complex, (I), was investigated. The binuclear copper(II) complex, Fig. 1, is situated about a centre of inversion and features two Cu II atoms bridged by four acetate groups. The Cu–O bond distances ranged from 1.941 (4) to 1.980 (4) Å (Table 1). The distorted square-pyramidal coordination environment for the Cu atom is completed by a pyridine-N atom and four carboxyl-O atoms of acetate anions. In the binuclear compound, the Cu ···Cu distance is 2.5997 (15) Å. In the crystal, the molecules are linked by weak C—H···O and C—H···N hydrogen bonds into three dimensional supramolecular architecture.

### 2. Experimental

2-Cyanopyridine (23.1233 g, 30 mmol) was added to a THF solution (60 ml) of Cu(OAc)_2_.H_2_O (1.9972 g, 5 mmol). The reaction mixture was stirred vigorously while refluxing for 48 h. The filtrate was slowly evaporated, the blue single crystals were obtained.

### 3. Refinement

H atoms were placed in calculated position with C—H = 0.93 (aromatic) and 0.96 Å (methyl), and refined in a riding mode with U_iso_(H) = 1.2U_eq_(C) for aromatic H atoms and 1.5U_eq_(C) for methyl H atom.

### Crystal data

**Table:**

[Cu_2_(C_2_H_3_O_2_)_4_(C_6_H_4_N_2_)_2_]	*F*(000) = 580
*M**_r_* = 571.48	*D*_x_ = 1.677 Mg m^−^^3^
Monoclinic, *P*2_1_/*c*	Mo *K*α radiation, λ = 0.71073 Å
Hall symbol: -P 2ybc	Cell parameters from 3034 reflections
*a* = 7.929 (5) Å	θ = 2.9–30.5°
*b* = 19.817 (12) Å	µ = 1.93 mm^−^^1^
*c* = 8.222 (5) Å	*T* = 140 K
β = 118.83 (2)°	Block, blue
*V* = 1131.8 (12) Å^3^	0.32 × 0.12 × 0.06 mm
*Z* = 2	

### Data collection

**Table:**

Bruker APEXII CCD diffractometer	2077 independent reflections
Radiation source: fine-focus sealed tube	1792 reflections with *I* > 2σ(*I*)
Graphite monochromator	*R*_int_ = 0.165
φ and ω scans	θ_max_ = 25.3°, θ_min_ = 1.0°
Absorption correction: multi-scan (*SADABS*; Bruker, 2001)	*h* = −9→9
*T*_min_ = 0.58, *T*_max_ = 0.89	*k* = −23→23
7361 measured reflections	*l* = −9→6

### Refinement

**Table:**

Refinement on *F*^2^	Primary atom site location: structure-invariant direct methods
Least-squares matrix: full	Secondary atom site location: difference Fourier map
*R*[*F*^2^ > 2σ(*F*^2^)] = 0.060	Hydrogen site location: inferred from neighbouring sites
*wR*(*F*^2^) = 0.159	H-atom parameters constrained
*S* = 1.07	*w* = 1/[σ^2^(*F*_o_^2^) + (0.0938*P*)^2^] where *P* = (*F*_o_^2^ + 2*F*_c_^2^)/3
2077 reflections	(Δ/σ)_max_ < 0.001
157 parameters	Δρ_max_ = 0.89 e Å^−^^3^
0 restraints	Δρ_min_ = −1.48 e Å^−^^3^

### Special details

**Table:**

Geometry. All esds (except the esd in the dihedral angle between two l.s. planes) are estimated using the full covariance matrix. The cell esds are taken into account individually in the estimation of esds in distances, angles and torsion angles; correlations between esds in cell parameters are only used when they are defined by crystal symmetry. An approximate (isotropic) treatment of cell esds is used for estimating esds involving l.s. planes.
Refinement. Refinement of F^2^ against ALL reflections. The weighted R-factor wR and goodness of fit S are based on F^2^, conventional R-factors R are based on F, with F set to zero for negative F^2^. The threshold expression of F^2^ > 2sigma(F^2^) is used only for calculating R-factors(gt) etc. and is not relevant to the choice of reflections for refinement. R-factors based on F^2^ are statistically about twice as large as those based on F, and R- factors based on ALL data will be even larger.

### Fractional atomic coordinates and isotropic or equivalent isotropic displacement parameters (Å^2^)

**Table:**

	*x*	*y*	*z*	*U*_iso_*/*U*_eq_	
Cu1	0.40864 (8)	0.05361 (2)	0.50591 (7)	0.0184 (3)	
N1	0.2510 (5)	0.14273 (19)	0.5328 (6)	0.0199 (8)	
N2	0.6530 (7)	0.2387 (2)	0.6344 (9)	0.0436 (14)	
O1	0.4618 (6)	0.08980 (17)	0.3114 (5)	0.0295 (8)	
O2	0.6269 (6)	−0.00139 (17)	0.3074 (5)	0.0283 (8)	
O3	0.3373 (5)	−0.08126 (18)	0.3035 (5)	0.0318 (8)	
O4	0.1766 (5)	0.00951 (17)	0.3166 (5)	0.0260 (8)	
C1	0.0735 (7)	0.1301 (2)	0.5040 (7)	0.0253 (11)	
H1	0.0229	0.0871	0.4645	0.030*	
C2	−0.0387 (7)	0.1775 (2)	0.5298 (7)	0.0270 (11)	
H2	−0.1624	0.1666	0.5070	0.032*	
C3	0.0343 (7)	0.2405 (2)	0.5895 (7)	0.0264 (11)	
H3	−0.0394	0.2731	0.6075	0.032*	
C4	0.2182 (8)	0.2557 (2)	0.6228 (7)	0.0261 (11)	
H4	0.2711	0.2982	0.6643	0.031*	
C5	0.3216 (7)	0.2053 (2)	0.5922 (7)	0.0201 (10)	
C6	0.5091 (8)	0.2214 (2)	0.6174 (8)	0.0303 (12)	
C7	0.5577 (7)	0.0559 (2)	0.2514 (7)	0.0214 (10)	
C8	0.5910 (9)	0.0865 (3)	0.1015 (8)	0.0320 (12)	
H8A	0.6835	0.0600	0.0860	0.048*	
H8B	0.6388	0.1317	0.1362	0.048*	
H8C	0.4719	0.0875	−0.0131	0.048*	
C9	0.1852 (8)	−0.0478 (2)	0.2547 (7)	0.0228 (10)	
C10	0.0014 (8)	−0.0787 (3)	0.1106 (8)	0.0324 (12)	
H10A	−0.0097	−0.0723	−0.0099	0.049*	
H10B	−0.1056	−0.0575	0.1144	0.049*	
H10C	0.0014	−0.1261	0.1349	0.049*	

### Atomic displacement parameters (Å^2^)

**Table:**

	*U*^11^	*U*^22^	*U*^33^	*U*^12^	*U*^13^	*U*^23^
Cu1	0.0166 (4)	0.0163 (3)	0.0162 (4)	0.0018 (2)	0.0029 (3)	−0.0010 (2)
N1	0.0151 (18)	0.0218 (18)	0.018 (2)	0.0014 (16)	0.0041 (16)	−0.0018 (16)
N2	0.029 (3)	0.028 (2)	0.079 (4)	−0.007 (2)	0.031 (3)	−0.014 (2)
O1	0.038 (2)	0.0219 (16)	0.031 (2)	0.0029 (16)	0.0185 (18)	0.0034 (15)
O2	0.034 (2)	0.0291 (18)	0.0237 (19)	0.0055 (16)	0.0161 (17)	0.0009 (15)
O3	0.0231 (18)	0.0252 (17)	0.034 (2)	−0.0019 (17)	0.0034 (17)	−0.0079 (16)
O4	0.0175 (17)	0.0260 (17)	0.0216 (19)	0.0029 (14)	−0.0010 (15)	−0.0027 (14)
C1	0.019 (2)	0.024 (2)	0.025 (3)	−0.005 (2)	0.005 (2)	−0.003 (2)
C2	0.019 (2)	0.028 (2)	0.031 (3)	0.000 (2)	0.010 (2)	0.001 (2)
C3	0.023 (3)	0.024 (2)	0.029 (3)	0.006 (2)	0.010 (2)	0.001 (2)
C4	0.028 (3)	0.020 (2)	0.029 (3)	0.003 (2)	0.013 (2)	0.000 (2)
C5	0.020 (2)	0.018 (2)	0.019 (2)	0.0016 (19)	0.007 (2)	0.0021 (18)
C6	0.034 (3)	0.018 (2)	0.042 (3)	0.004 (2)	0.021 (3)	−0.002 (2)
C7	0.019 (2)	0.022 (2)	0.016 (2)	−0.003 (2)	0.004 (2)	−0.0023 (18)
C8	0.040 (3)	0.030 (3)	0.023 (3)	−0.006 (2)	0.013 (2)	0.001 (2)
C9	0.022 (3)	0.022 (2)	0.014 (2)	−0.003 (2)	0.001 (2)	0.0004 (19)
C10	0.024 (3)	0.029 (3)	0.026 (3)	−0.005 (2)	−0.003 (2)	0.001 (2)

### Geometric parameters (Å, º)

**Table:**

Cu1—O1	1.975 (4)	C1—H1	0.9300
Cu1—O2^i^	1.980 (4)	C2—C3	1.366 (7)
Cu1—O3^i^	1.941 (4)	C2—H2	0.9300
Cu1—O4	1.952 (4)	C3—C4	1.380 (7)
Cu1—N1	2.235 (4)	C3—H3	0.9300
Cu1—Cu1^i^	2.5997 (15)	C4—C5	1.389 (7)
N1—C1	1.334 (6)	C4—H4	0.9300
N1—C5	1.352 (6)	C5—C6	1.435 (7)
N2—C6	1.133 (7)	C7—C8	1.507 (7)
O1—C7	1.279 (6)	C8—H8A	0.9600
O2—C7	1.248 (6)	C8—H8B	0.9600
O2—Cu1^i^	1.980 (4)	C8—H8C	0.9600
O3—C9	1.261 (7)	C9—C10	1.496 (7)
O3—Cu1^i^	1.941 (4)	C10—H10A	0.9600
O4—C9	1.259 (6)	C10—H10B	0.9600
C1—C2	1.379 (7)	C10—H10C	0.9600
			
O3^i^—Cu1—O4	169.17 (14)	C2—C3—C4	119.6 (5)
O3^i^—Cu1—O1	90.43 (17)	C2—C3—H3	120.2
O4—Cu1—O1	90.23 (16)	C4—C3—H3	120.2
O3^i^—Cu1—O2^i^	90.11 (17)	C3—C4—C5	117.9 (5)
O4—Cu1—O2^i^	87.28 (16)	C3—C4—H4	121.1
O1—Cu1—O2^i^	169.40 (14)	C5—C4—H4	121.1
O3^i^—Cu1—N1	96.27 (15)	N1—C5—C4	123.2 (4)
O4—Cu1—N1	94.34 (15)	N1—C5—C6	118.4 (4)
O1—Cu1—N1	98.10 (15)	C4—C5—C6	118.4 (4)
O2^i^—Cu1—N1	92.35 (14)	N2—C6—C5	175.1 (6)
O3^i^—Cu1—Cu1^i^	83.16 (11)	O2—C7—O1	125.0 (4)
O4—Cu1—Cu1^i^	86.12 (11)	O2—C7—C8	116.7 (4)
O1—Cu1—Cu1^i^	85.76 (11)	O1—C7—C8	118.3 (4)
O2^i^—Cu1—Cu1^i^	83.80 (10)	C7—C8—H8A	109.5
N1—Cu1—Cu1^i^	176.10 (10)	C7—C8—H8B	109.5
C1—N1—C5	117.0 (4)	H8A—C8—H8B	109.5
C1—N1—Cu1	115.2 (3)	C7—C8—H8C	109.5
C5—N1—Cu1	127.4 (3)	H8A—C8—H8C	109.5
C7—O1—Cu1	121.3 (3)	H8B—C8—H8C	109.5
C7—O2—Cu1^i^	124.0 (3)	O4—C9—O3	125.1 (5)
C9—O3—Cu1^i^	124.7 (3)	O4—C9—C10	117.9 (5)
C9—O4—Cu1	120.8 (3)	O3—C9—C10	117.0 (4)
N1—C1—C2	123.3 (5)	C9—C10—H10A	109.5
N1—C1—H1	118.4	C9—C10—H10B	109.5
C2—C1—H1	118.4	H10A—C10—H10B	109.5
C3—C2—C1	119.1 (4)	C9—C10—H10C	109.5
C3—C2—H2	120.5	H10A—C10—H10C	109.5
C1—C2—H2	120.5	H10B—C10—H10C	109.5

Symmetry code: (i) −*x*+1, −*y*, −*z*+1.

### Hydrogen-bond geometry (Å, º)

**Table:**

*D*—H···*A*	*D*—H	H···*A*	*D*···*A*	*D*—H···*A*
C2—H2···N2^ii^	0.93	2.60	3.196 (9)	122
C8—H8*B*···N2^iii^	0.96	2.57	3.491 (8)	160
C10—H10*A*···O4^iv^	0.96	2.54	3.381 (7)	147

Symmetry codes: (ii) *x*−1, *y*, *z*; (iii) *x*, −*y*+1/2, *z*−1/2; (iv) −*x*, −*y*, −*z*.
